# Comparison of *Toxoplasma gondii* IgG avidity Architect and Vidas assays with the estimated date of infection in pregnant women

**DOI:** 10.1051/parasite/2016056

**Published:** 2016-10-20

**Authors:** Aurélie Smets, Thomas Fauchier, Grégory Michel, Pierre Marty, Christelle Pomares

**Affiliations:** 1 Laboratoire de Biologie Médicale, Centre Hospitalier de la Dracénie 83300 Draguignan France; 2 Service de Santé Publique, Unité EVARISQ, CHU Poitiers 86021 Poitiers France; 3 Université de Nice Sophia Antipolis, Faculté de Médecine 06107 Nice France; 4 INSERM U1065, Centre Méditerranéen de Médecine Moléculaire, C3M, Toxines Microbiennes dans la relation hôte-pathogènes 06200 Nice France; 5 Service de Parasitologie-Mycologie, Centre Hospitalier Universitaire de Nice 06202 Nice France

**Keywords:** *Toxoplasma gondii*, IgG avidity, Date of infection, Pregnant women, *Toxoplasma gondii* IgG ratio

## Abstract

A maternal *Toxoplasma gondii* infection during pregnancy is a risk for congenital infection through maternal-fetal transplacental transmission. Estimation of the date of infection is of the utmost importance for management and treatment recommendations. In this setting, IgG avidity has been shown to be useful as high avidity rules out an infection dating less than 4 months. The estimated date of infection can also be obtained by the ratio of *T. gondii* IgG titers measured by the Vidas (bioMérieux) assay versus *T. gondii* IgG titers measured by the Architect (Abbott Laboratories) test, together with *T. gondii* IgM and IgA antibody responses. In this study, using 117 serum samples from pregnant women, we compared the IgG avidity values obtained by Architect and Vidas with the presumed date of *T. gondii* infection established by the *T. gondii* IgG ratio of IgG Vidas and IgG Architect plus the IgM and IgA results. To date, IgG avidity Vidas seems to exhibit better performance than Architect. For both assays, gray zone results were most likely obtained from patients infected more than 4 months before sampling. These data should be taken into account for a possible reconsideration of the interpretation of avidity results in the gray zone.

## Abstract (fr)

Une primo infection maternelle par *Toxoplasma gondii* en cours de grossesse représente un risque d'infection congénitale par passage transplacentaire du parasite. Il est important d’estimer la date de cette primo infection car les recommandations de prise en charge et de traitement varient en fonction de cette estimation. L’avidité des IgG est particulièrement utile puisqu’elle permet, en cas d’avidité élevée d’exclure une infection datant de moins de quatre mois. L’estimation de la date de l'infection peut également être obtenue par le rapport des valeurs des IgG mesurées sur Vidas (bioMérieux) par rapport à ceux mesurés sur Architect (Abbott Laboratories) en tenant en compte aussi des valeurs des IgM et IgA anti *T. gondii*. Dans cette étude, en utilisant 117 échantillons de sérum de femmes enceintes, nous avons comparé les valeurs des avidités sur Architect et Vidas avec la date présumée de l'infection établie par le rapport d'IgG Vidas/IgG Architect plus les résultats des IgM et IgA. Dans notre étude, l’avidité Vidas semble avoir de meilleures performances que l’avidité Architect. Pour les deux tests, une avidité en zone grise est le plus souvent trouvée chez des patientes infectées plus de quatre mois avant la date de prélèvement. Ces données devraient être prises en compte lors de l’interprétation des résultats de l’avidité dans la zone grise.

## Introduction

*Toxoplasma gondii* is an obligate intracellular protozoan parasite that infects most species of warm-blooded animals, including humans. Infection by *T. gondii* is mainly acquired by ingestion of undercooked infected meat and through food or water that have been in contact with oocysts present in felines feces. A primary maternal infection acquired during pregnancy can lead to a risk of congenital infection. This risk is moderate (4–14%) if maternal infection occurs in the first trimester of pregnancy and higher (70–80%) if it occurs in the third trimester [14]. The severity of congenital infection is higher if fetal infection occurs earlier in pregnancy (intrauterine fetal death, intracranial calcifications, and hydrocephalus) [5]. Infections around birth are frequently asymptomatic [5].

Since 1978 in France, prevention of congenital infection by *T. gondii* has been based on serological identification and follow-up of pregnant women (*décret République Française* n°78-396 17 March 1978) [20]. Following the diagnosis of pregnancy, *T. gondii* serologies are performed monthly until delivery in seronegative women. Ideally, the *T. gondii* immune status of pregnant women should be established before pregnancy. However, in most of cases, the first serological screening is performed during the first trimester. Presence of specific *T. gondii* IgG without *T. gondii* IgM is consistent with chronic infection. Absence of IgG and IgM-specific antibodies leads to monthly follow-up until delivery. The simultaneous detection of IgG and IgM in the first serum sample requires additional testing to estimate the date of infection since IgM antibodies may also persist for up to 18 months or longer post infection [2, 6, 10, 18, 19]*.* In this setting, to rule out recent infection, a *T. gondii* avidity assay has been proven to be useful as high avidity rules out primary infection occurring in at least the preceding 4 months [1, 3, 8, 13, 19, 23]. In addition, comparison of *T. gondii* IgG results obtained from two serological methods using different antigen targets (predominantly *T. gondii* membrane antigens or predominantly cytoplasmic antigens) has also been proven to be a reliable method to estimate the date of infection [6, 12]. Lower *T. gondii* IgG titers against cytoplasmic antigens as compared to those observed against membrane antigens are in favor of a recently acquired infection [12]. The ratio of two IgG assays using different antigen targets has been validated and used routinely in our Laboratory since the first publication by Marty and Le Fichoux [12]. The IgG Architect (Abbott Laboratories) assay is positive earlier than the IgG Vidas (bioMérieux) assay during acute infection [6, 15]. In addition, the ratio *T. gondii* IgG Vidas divided by IgG Architect (IgG Vidas/IgG Architect) allows estimation of the date of infection [6, 12]. A ratio above one is in favor of an infection older than 4 months, whereas a ratio below one is most likely related to acute infection [12]. The aim of this study was to compare the estimated date of infection obtained by the IgG ratio described above with the estimated date of infection obtained by the *T. gondii* avidity assays Architect and Vidas.

## Materials and methods

### Serum specimens

Routine screening of *T. gondii* IgG and IgM in the Laboratory of Parasitology-Mycology of University Hospital of Nice (France) is performed on the Architect platform (Abbott Laboratories, Wiesbaden, Germany). In case of positive IgM, serum was prospectively selected for the study and the following tests were performed: IgG Vidas (bioMérieux, Marcy l’Etoile, France), *T. gondii* IgA (Platelia BioRad), avidity Architect and Vidas. A total of 117 consecutive sera from 117 healthy pregnant women were tested.

### Ethical aspects

The study was approved by the local Ethics Committee (*Comité de protection des personnes* CHU Nice, France).

### Interpretation of results

Positive cut-off values used to interpret the results of serological assays were those recommended by the manufacturers: IgG Architect ≥ 3 IU/mL; IgG Vidas ≥ 8 IU/mL; IgM Architect index ≥ 0.6, and IgA Platelia BioRad index ≥ 1. According to the manufacturer’s instructions, results of avidity assays were interpreted as high, gray zone and low, respectively: Architect avidity ≥ 60%, 50–59.9%, < 50%; Vidas avidity ≥ 30%, 20%–30%, < 20% [22].

Sera were classified in three groups according to the estimated date of maternal infection obtained by the ratio IgG Vidas/IgG Architect in addition to the IgM and IgA serological tests results [6, 12]. Estimation of the date of infection was performed as followed:

- Maternal acute infection acquired within 4 months before the date of sampling – group AI < 4 (*n* = 17): IgG Vidas/IgG Architect ratio < 1 and positive IgM Architect ± positive IgA.
- Maternal chronic infection older than 4 months to be confirmed because of positive IgA – group CI > 4 TBC [to be confirmed] (*n* = 9): ratio IgG Vidas/IgG Architect > 1, positive IgM and positive IgA. Indeed, positive IgA can be present during acute infection but also during chronic infection older than 4 months [16, 21].
- Maternal chronic infection older than 4 months – group CI > 4 (*n* = 91): IgG Vidas/IgG Architect ratio > 1, positive IgM, negative IgA.

The two avidity Architect and Vidas assays were compared for their correlation with the estimated date of infection defined by the above criteria.

### Data analysis

The VassarStats website for Statistical Computation was used for the statistical analyses. Cohen’s kappa coefficient (CKC) was used to compare the results given by each *T. gondii* avidity assay (Architect and Vidas) to the estimated date of infection as described above. The more the agreement between the two avidity assays results and estimated date of infection increases, the more the kappa coefficient increases. A kappa of 1 corresponds to perfect agreement, whereas kappa of 0 is an agreement by chance. Kappa coefficients between 0.81 and 0.99 is almost perfect, between 0.61 and 0.80 is substantial agreement, and between 0.41 and 0.60 is moderate agreement [4, 23].

## Results

The avidity Architect and Vidas assays were compared to the estimated date of infection (Table 1). For the 17 sera classified in group AI < 4, the low avidity values were in agreement with the estimated date of infection expect for one Vidas avidity classified in the gray zone. For the sera classified in group CI > 4 TBC, a low avidity was found with Architect and Vidas in 8 and 5 out of 9 sera, respectively. Among the CI > 4 patient group (*n* = 91), high IgG avidity was found in 69.2% (*n* = 63) and 83.5% (*n* = 76) of Architect and Vidas avidity assays, respectively (Table 1). Among the patients in groups AI < 4 and CI > 4 TBC (*n* = 26), a low avidity test result was found in 96.2% (*n* = 25) and 80.8% (*n* = 21) of Architect and Vidas assays, respectively (Table 1). However, it must be remembered that neither Architect nor Vidas avidities are recommended for the diagnosis of acute toxoplasmosis. If we consider for both tests that avidity values in the gray zone rule out recently acquired infection, the percentages of accurate conclusions (CI > 4, *n* = 91) would have been 86.8% (*n* = 79) and 93.4% (*n* = 85) for Architect and Vidas avidity assays, respectively.

**Table 1.** Avidity data from Architect and Vidas assays according to the estimated date of infection established by IgG Vidas/IgG Architect ratio and IgM and IgA tests results.

	Architect avidity			Vidas avidity		
	High *n* = 63	Gray zone *n* = 17	Low *n* = 37	High *n* = 76	Gray zone *n* = 14	Low *n* = 27
Group AI < 4, *n* = 17	0	0	17	0	1	16
Group CI > 4 TBC, *n* = 9	0	1	8	0	4	5
Group CI > 4, *n* = 91	63	16	12	76	9	6

Group AI < 4: maternal infection acquired within 4 months before date of sampling; Group CI > 4 TBC: chronic maternal infection older than 4 months to be confirmed (TBC); Group CI > 4: chronic maternal infection older than 4 months.

In order to evaluate the conditions for a better agreement between avidities (Architect and Vidas) and estimated date of infection (obtained from the IgG Vidas/IgG Architect ratio), sera with values in the gray zone and those in group CI > 4 TBC were redistributed as detailed in Table 2. For each of the redistributions of sera, CKC and the number of discrepant results with the estimated date of infection were calculated (Table 2). The best correlation with the estimated date of infection was obtained with the Vidas avidity assay when sera in the gray zone and sera in group CI > 4 TBC were excluded (Table 2). However, this result is biased due to exclusion of sera that were difficult to interpret. Taking into account all samples, Architect and Vidas avidities have the best correlation with the estimated date of infection when sera in the gray zone are considered as if they were in the high avidity group and sera in group CI > 4 TBC are considered as belonging to group AI < 4 (Table 2). This observation means that a gray zone avidity could be used to rule out an infection dating less than 4 months.

**Table 2.** Agreements between avidity tests results and estimated date of infection after redistribution of sera with values in the gray zone and belonging to group CI > 4 TBC.

Redistribution of sera	Groups	Architect avidity				Vidas avidity			
		High	Low	Kappa coefficient	Number of discrepant results with the estimated date of infection	High	Low	Kappa coefficient	Number of discrepant results with the estimated date of infection
Exclusion of sera in gray zone avidity and in group CI > 4 TBC	Group AI < 4	0	17	0.660	12	0	16	**0.805**	6
	Group CI > 4	63	12			76	6		
Gray zone avidity in high avidity sera and group CI > 4 TBC in CI > 4 group	Group AI < 4	0	17	0.538	20	1	16	0.668	12
	Group CI > 4 bis: CI > 4 TBC + CI > 4	80	20			89	11		
Gray zone avidity in high avidity sera and group CI > 4 TBC in group AI < 4	Group AI < 4 bis: CI > 4 TBC + AI < 4	1	25	**0.721**	13	5	21	**0.712**	11
	Group CI > 4	79	12			85	6		
Gray zone avidity in low avidity sera and group CI > 4 TBC in group AI < 4	Group AI < 4 bis: CI > 4 TBC + AI < 4	0	26	0.500	28	0	26	0.693	15
	Group CI > 4	63	28			76	15		

*Notes*. In the first redistribution (exclusion of samples in gray zone avidity and samples belonging to group CI > 4 TBC), 25 and 19 sera for Architect and Vidas avidity assays were excluded, respectively. In bold, the three best CKC obtained depending the exclusion/redistribution of sera.

## Discussion

This study compared two avidity assays with the estimated date of infection obtained by the IgG Vidas/IgG Architect ratio, associated with the results of IgM and IgA. The estimated date of infection is particularly important when serum samples are positive for IgG and IgM during the first trimester of pregnancy. This is because the presence of IgM does not always reflect acute infection. Some IgM results could be false positives as serological testing for *T. gondii* infection is not uniformly consistent in commercial kits [9]. Moreover, significant titers of IgM could persist for a long period of time after acute infection, leading to interpretation difficulties [2, 7, 10, 18, 19]. During pregnancy, avidity assays can be performed to differentiate an infection dating more than 4 months from a recent one. As specified in the manufacturer’s instructions, avidity is an exclusion assay: a high avidity index is a strong indication of primary infection dating more than 4 months [1, 3, 8, 13, 19, 23]. Consensus exists that a high avidity result obtained in the first trimester of pregnancy rules out acquired infection during gestation [22]. This information is of the utmost importance since unnecessary treatment and follow-up can be avoided.

In this study, two commercially available automated avidity immunoassays were evaluated using sera from pregnant women from Nice, France. For both assays, a high avidity index always correlated with primary infection dating back more than 4 months, as established by the IgG Vidas/IgG Architect ratio. In our cohort, there were no unexpected results as in the group of *T. gondii* recent infection, no high avidity was found. However, among the 91 sera from patients with infection dating more than 4 months, Architect and Vidas avidities were high in 69.2% and 83.5%, respectively. This study confirms that IgG avidity Vidas had the best performance for the diagnosis of an infection dating more than 4 months [22]. This discrepancy between both avidity assays could be explained by differences in avidity maturation and/or by the use of recombinant antigens for Architect avidity [11, 22]. In addition, it has already been demonstrated for Vidas avidity, that an avidity higher than the gray zone threshold (20%) could safely be used to rule out a recently acquired infection of less than 4 months [7]. In this setting, efficacy of Vidas avidity (93.4% of accuracy) was better than Architect avidity (86.8%) when compared to our estimated date of infection.

The presumed date of infection was estimated by comparison of two serological techniques using different antigenic targets. As already reported, the kinetics of the two assays are different: IgG Architect become positive earlier than IgG Vidas [6, 15]. Therefore, the IgG Vidas/IgG Architect ratio allowed us to reliably estimate the date of infection [6, 12]. Excluding for both assays gray zone avidity and group CI > 4 TBC sera, an almost perfect agreement for Vidas avidity (CKC = 0.805) was found, whereas the agreement between Architect avidity and estimated date of infection was substantial (CKC = 0.660) (Table 2). For both tests, agreement fails if sera in the gray zone are included in high avidity sera and group CI > 4 TBC is included in group CI > 4 (Group CI > 4 bis: CI > 4 TBC + CI > 4). However, close agreement was restored when group CI > 4 TBC was included in group AI < 4 (Group AI < 4 bis: CI > 4 TBC + AI < 4) and sera in the gray zone were included in the high avidity sera. These results suggested that in group CI > 4 TBC, samples were predominantly from patients with acute infection. Conversely, when gray zone avidity sera were included in the low avidity sera and group CI > 4 TBC in group AI < 4 (Group AI < 4 bis: CI > 4 TBC + AI < 4), kappa coefficients for both tests decreased (CKC Vidas = 0.693 and Architect = 0.500) (Table 2). These results highlighted the fact that for both tests, the classification in gray zone avidity strongly suggested an infection older than 4 months. When the conclusion was “chronic maternal infection older than 4 months to be confirmed” (CI > 4 TBC), a further serological control has to be done, as on the first serum, results of avidity would have been in favor of acute infection [7]. In this case, the kinetics of the IgG titers on the previous and follow-up serum samples, run at the time, often allowed us to estimate the date of infection. This occurrence is rare and most often, the date of infection can be obtained on the first serum sample [17].

In our study, we found that Vidas avidity exhibited better performance than Architect avidity to rule out infection that occurred less than 4 months before. For both avidity assays, when comparing with our estimated date of infection, a better agreement was observed when gray zone avidity results were redistributed in high avidity, leading to the conclusion that gray zone results were most likely obtained from patients infected more than 4 months before sampling. These data could be taken into account for a possible reconsideration of the interpretation of avidity results in the gray zone.
